# Synthetic Polymers for Biomedical Applications

**DOI:** 10.1155/2018/7158621

**Published:** 2018-04-24

**Authors:** Qiang Wei, Nan-Nan Deng, Junling Guo, Jie Deng

**Affiliations:** ^1^Max Planck Institute for Medical Research, Heidelberg, Germany; ^2^Harvard University, Cambridge, MA, USA; ^3^University of Freiburg, Freiburg, Germany

Polymers have been emerging to be the cornerstones for therapeutic applications as well as the largest and versatile class of biomaterials. Synthetic polymers can be designed and synthesized with a broad variety of structures and appropriate physical and chemical properties, which are of increasing interest in a wide range of biomedical applications as diverse as tissue engineering, drug delivery, therapeutics, diagnostics, and so on.

Since the last decade, the methods of polymer synthesis, processing, and characterization are developing rapidly, which bring both challenges and opportunities to design novel polymeric biomaterials as well as to understand the biological behaviors between biological systems and polymeric materials. Therefore, we launch this special issue, including two review articles and four research articles, to summarize the application of synthetic polymers in biomedical engineering and to illustrate the new development of polymeric biomaterials.

One review article “Strain and Vibration in Mesenchymal Stem Cells” focuses on the effect of various culture conditions and strain or vibration parameters to review the response of mesenchymal stem cells to vibration and cyclic tension and then discuss how polymer scaffolds influence cell response to vibration and strain. The other review article “Scaffolds for Pelvic Floor Prolapse: Logical Pathways” highlights the recently developed macroporous monofilament meshes and electrospinning emerged method, which may fill the gap in the market to treat pelvic organ prolapse. These two review articles indicate the importance of synthetic polymer scaffold in basic research and therapeutics, respectively.

The research articles in this issue extend the application of synthetic polymers in both basic research and therapeutics. Synthetic poly(lactic-co-glycolic acid) (PLGA) is widely considered as a base material for biomedical applications due to its good biocompatibility and degradability. In the article “Influence of Processing Conditions on the Mechanical Behavior and Morphology of Injection Molded Poly(lactic-co-glycolic acid) 85:15,” an overview is provided among processing conditions, morphology, and mechanical property relationship of injection molded PLGA. Based on the study of mechanics, PLGA is further processed by injection molding as craniofacial bioresorbable medical devices in the article “Effect of Injection Molding Melt Temperatures on PLGA Craniofacial Plate Properties during* In Vitro* Degradation.” The mechanical and physicochemical properties of the PGA plates are evaluated in detail during in vitro degradation. G. Rijal et al. fabricated 3D porous scaffolds via PLGA and another biodegradable synthetic polymer polycaprolactone (PCL) in the article “Application of Synthetic Polymeric Scaffolds in Breast Cancer 3D Tissue Cultures and Animal Tumor Models.” It has proven that cancer cells grown on 3D polymeric scaffolds exhibit distinct survival, morphology, and proliferation compared to those on 2D polymeric surfaces. Tumor models produced via these 3D scaffolds have obvious advantages in anticancer drug screening, which can facilitate the observations of cancer biomarker expression, molecular regulation of cancer progression, and drug efficacies across tumors at similar sizes and developmental stages. Polyethylene glycol (PEG) is another one of the most commonly used synthetic polymers for biomaterials. In the article “Efficient Self-Assembly of mPEG End-Capped Porous Silica as a Redox-Sensitive Nanocarrier for Controlled Doxorubicin Delivery,” porous nanosilica particles are modified with PEG shell via disulfide bridges and supramolecular interaction for drug delivery, with benefits of enhanced drug loading capacity and decreased risk of systemic toxicity.

In summary, this special issue connects the synthetic polymers to biomaterials science and engineering. We sincerely hope that the readers enjoy reading the presented original research work in this special issue and get inspired for their future studies.

